# Naringenin as a neurotherapeutic agent in Alzheimer’s disease: epigenetic signatures, gut microbiota alterations, and molecular neuroprotection

**DOI:** 10.3389/fnagi.2025.1647967

**Published:** 2025-08-15

**Authors:** Zhenzhen Lai, Long Ke, Wei Zhao

**Affiliations:** ^1^Department of Neurology, Tiantai People’s Hospital of Zhejiang Province, Tiantai Branch of Zhejiang Provincial People’s Hospital, Hangzhou Medical College, Taizhou, Zhejiang, China; ^2^Department of Health Examination, The Third Affiliated Hospital of Shanghai University, Wenzhou No. 3 Clinical Institute Affiliated to Wenzhou Medical University, Wenzhou People's Hospital, Wenzhou, China; ^3^Department of Neurology, Taizhou Integrated Traditional Chinese and Western Medicine Hospital, Wenling, Zhejiang, China

**Keywords:** naringenin, Alzheimer’s disease, epigenetic, gut microbiota, molecular neuroprotection

## Abstract

Alzheimer’s disease (AD) remains a major neurodegenerative disorder characterized by progressive cognitive decline, amyloid-*β* (Aβ) aggregation, tau pathology, oxidative stress, and chronic neuroinflammation. In recent years, the dietary flavonoid naringenin, abundant in citrus fruits, has gained attention as a multi-target neuroprotective agent with potential application in AD therapy. Preclinical studies demonstrate that naringenin exhibits robust antioxidant activity, notably through activation of the nuclear factor erythroid 2-related factor 2 (Nrf2)/antioxidant response element (ARE) signaling pathway, which reduces ROS and preserves mitochondrial integrity. Furthermore, naringenin upregulates AMPK-mediated autophagy, aiding in the clearance of toxic Aβ peptides and promoting neuronal survival. Inflammatory cascades are significantly downregulated following naringenin treatment. Additionally, naringenin modulates estrogen receptor and PI3K/Akt signaling, contributing to enhanced neuronal viability and reduced apoptosis. Notably, its ability to inhibit acetylcholinesterase suggests promise for restoring cholinergic neurotransmission. Despite these benefits, naringenin’s poor solubility and limited oral bioavailability hinder clinical translation. To address these challenges, advanced nanocarrier-based delivery systems have been engineered to facilitate blood–brain barrier penetration and sustained brain targeting, markedly improving cognitive outcomes in animal models. Safety profiles in rodents indicate low toxicity at therapeutic doses, reinforcing its viability as a candidate compound. This review highlights the multifaceted mechanisms and delivery strategies of naringenin in AD, and underscores the need for well-designed clinical trials to confirm its efficacy and safety in humans.

## Graphical Abstract

**Figure:**
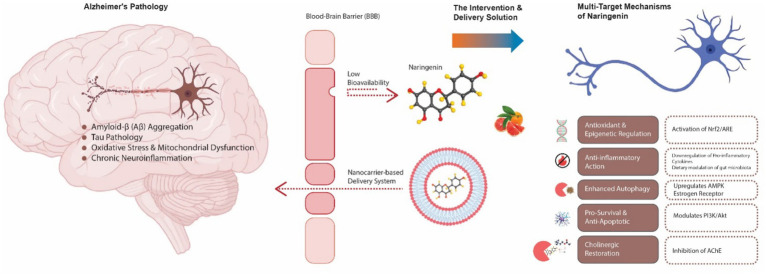
The effects of naringenin on epigenetic signatures, gut microbiota alterations, and molecular neuroprotection in in Alzheimer’s disease.

## 1 Introduction

AD is currently the most common form of dementia, contributing to 60–80% of all dementia cases globally (Livingston et al., 2020). As a progressive neurodegenerative disorder, AD is primarily characterized by memory deterioration, cognitive impairment, and behavioral changes that severely affect a person’s ability to carry out daily tasks (American Psychiatric Association, 1980). The pathological hallmarks of this condition include extracellular deposition of amyloid-*β* (Aβ) plaques, intracellular accumulation of hyperphosphorylated tau protein in the form of neurofibrillary tangles (NFTs), widespread neuronal death, synaptic dysfunction, and chronic neuroinflammation. Despite over a century of research since AD was first described in 1907, the exact etiology remains complex and multifactorial, involving genetic, biochemical, environmental, and lifestyle-related contributors (Farlow, 1998; Mattson, 2004). The global burden of AD is rapidly escalating. According to current projections, the number of individuals affected by dementia could exceed 150 million by 2050 if no effective treatment or preventive strategy is developed. In the United States alone, Alzheimer’s is ranked as the sixth leading cause of death and the fifth leading cause among individuals aged 65 and older. It is estimated that a new case of AD arises every 33 s, with nearly one million new diagnoses expected annually by mid-century (Alzheimer's Association, 2016). Moreover, the disease not only contributes to high mortality and disability rates but also imposes a severe financial burden on healthcare systems and society. The cost of care rises dramatically as the disease progresses, and the indirect costs—particularly those related to informal caregiving and productivity loss—represent a significant proportion of total expenditures. The economic impact is predicted to reach two trillion US dollars worldwide by 2030, underscoring the urgent need for more effective management strategies (Tay et al., 2024; Mathers and Loncar, 2006).

Presently, treatment options for AD are limited and largely symptomatic. Available pharmacological interventions, including acetylcholinesterase inhibitors and NMDA receptor antagonists, offer only temporary cognitive stabilization without halting or reversing disease progression (Marksteiner and Schmidt, 2004). Furthermore, these medications are often associated with variable efficacy and adverse effects. One of the major challenges in developing disease-modifying therapies is the difficulty of delivering drugs effectively across the blood–brain barrier (BBB), a highly selective interface that limits the entry of many therapeutic agents into the central nervous system (CNS) (Pardridge, 2020). The failure of numerous drug candidates in late-stage clinical trials further highlights the need for alternative approaches, particularly in the early stages of the disease when preventive strategies are more likely to be effective. Given these limitations, there has been growing interest in the neuroprotective potential of naturally derived compounds, particularly polyphenols, which are abundant in fruits, vegetables, and medicinal plants. Among them, flavonoids—a subclass of polyphenols—have attracted significant attention due to their diverse pharmacological activities, including antioxidant, anti-inflammatory, and anti-apoptotic effects (Kovacsova et al., 2010; Spagnuolo et al., 2016). Naringenin, a flavanone commonly found in citrus fruits such as grapefruits, oranges, and lemons, has emerged as a particularly promising candidate. It exists predominantly in the aglycone form or as glycosides like naringin and narirutin, and its structure enables favorable interaction with cellular targets associated with oxidative stress, inflammation, and amyloid metabolism (Morita et al., 2020; Azuma et al., 2020).

Naringenin (2,3-dihydro-5,7-dihydroxy-2-(4-hydroxyphenyl)-4H-1-benzopyran-4-one) is a hydrophobic molecule with a molecular weight of 272.25 g/mol (Motallebi et al., 2022; Zaidun et al., 2018). Despite its limited water solubility, studies have shown that naringenin can cross the BBB, likely via passive diffusion or active transport mechanisms, depending on its physicochemical properties and interactions with specific transporter proteins (Stasiłowicz-Krzemień et al., 2022; Youdim et al., 2004). Once in the CNS, naringenin has been shown to accumulate in brain regions associated with cognition and memory. Its ability to modulate multiple signaling pathways makes it a valuable compound for targeting the multifaceted pathogenesis of AD. Preclinical research has demonstrated that naringenin exhibits neuroprotective effects through several interrelated mechanisms. One of its most widely studied properties is its antioxidant activity. Naringenin reduces intracellular levels of reactive oxygen species (ROS) and enhances the activity of endogenous antioxidant enzymes such as superoxide dismutase (SOD), catalase (CAT), and glutathione peroxidase (GPx). In AD models, oxidative stress is known to exacerbate Aβ aggregation and tau hyperphosphorylation, leading to neuronal injury. By mitigating oxidative damage and preserving mitochondrial function, naringenin can help maintain neuronal integrity and viability (Wang et al., 2017; Yang et al., 2025). Chronic neuroinflammation, primarily mediated by activated microglia and astrocytes, plays a central role in AD progression (Wang et al., 2023). Naringenin has been shown to inhibit the expression of pro-inflammatory cytokines such as interleukin-1 beta (IL-1*β*), Tumor necrosis factor-alpha (TNF-*α*) and interleukin-6 (IL-6), as well as suppress the activation of NF-κB and MAPK signaling cascades (Yang et al., 2021). These actions help dampen the inflammatory response and reduce neuronal apoptosis. Naringenin treatment has led to decreased glial activation and improved cognitive performance (Wu et al., 2016). Furthermore, naringenin influences key processes in amyloid metabolism. It can downregulate the expression of amyloid precursor protein (APP) and β-secretase (BACE1), leading to reduced formation of Aβ peptides (Lee et al., 2018). Some studies also suggest that naringenin and its derivatives inhibit Aβ aggregation and promote its clearance via enhanced autophagy. Additionally, by reducing glycogen synthase kinase-3β (GSK-3β) activity, naringenin indirectly limits tau hyperphosphorylation, thereby preventing the formation of toxic NFTs (Mokhtar et al., 2024; Lima et al., 2023; Zhang et al., 2018).

Beyond these molecular targets, naringenin contributes to the preservation of synaptic function. It modulates neurotrophic signaling pathways, including BDNF–TrkB-CREB, and enhances long-term potentiation (LTP), which is crucial for memory consolidation. Naringenin has also demonstrated the ability to improve learning and memory in animal models of AD, supporting its role in functional recovery (Nemati et al., 2025; Alijani et al., 2025). Emerging evidence also points to the relevance of the gut–brain axis in AD pathophysiology. Dysbiosis of the gut microbiota has been linked to neuroinflammation and cognitive impairment (Megur et al., 2020). Naringenin, with its prebiotic-like effects, has been found to reshape the gut microbial composition, increase the production of beneficial short-chain fatty acids (SCFAs), and reduce systemic inflammation. These actions may indirectly contribute to neuroprotection and cognitive resilience (Cichon et al., 2025; Pinho-Ribeiro et al., 2016). Taken together, these findings position naringenin as a multifaceted compound with the potential to target several pathological features of AD simultaneously. This review aims to comprehensively examine the mechanistic pathways through which naringenin exerts its protective effects in AD, including its antioxidant, anti-inflammatory, anti-amyloidogenic, mitochondrial-stabilizing, synaptic-preserving, and microbiota-modulating actions. Understanding these mechanisms may offer new perspectives on how dietary flavonoids like naringenin can be leveraged in the prevention and adjunctive management of AD.

## 2 Pathophysiological hallmarks of AD

AD is characterized by a progressive decline in cognitive function, rooted in a complex cascade of molecular and cellular disturbances. One of the earliest and most studied events in AD pathology is the accumulation of A*β* peptides. These peptides are generated through the cleavage of APP by β-secretase and *γ*-secretase enzymes. When Aβ monomers aggregate into soluble oligomers, they exert toxic effects on neurons, disrupting cellular signaling and synaptic communication well before they form extracellular plaques (Selkoe and Hardy, 2016; Ahmad et al., 2019). These oligomers are now recognized as critical contributors to early synaptic dysfunction and memory impairment in AD (Haass and Selkoe, 2007; Meng et al., 2019). In parallel, tau protein—normally responsible for stabilizing microtubules in neurons—undergoes abnormal hyperphosphorylation in AD. This modification diminishes its ability to bind to microtubules and causes the formation of intracellular aggregates known as NFTs. The accumulation of these tangles follows a stereotypical progression through brain regions involved in memory and cognition, such as the hippocampus and entorhinal cortex. As tau pathology spreads, it correlates closely with the severity of clinical symptoms, reinforcing its central role in disease progression (Goedert et al., 1989; Wegmann et al., 2021). Oxidative stress is another major pathological feature in AD and is detectable in brain tissue even at early stages of the disease. Damaged mitochondria—key producers of cellular energy—become less efficient in generating ATP and more prone to releasing ROS. This oxidative imbalance damages lipids, proteins, and nucleic acids, leading to neuronal injury and contributing to disease advancement. Mitochondrial dysfunction not only impairs energy metabolism but also promotes further Aβ accumulation and tau pathology, establishing a harmful feedback loop (Castellani et al., 2002; Wang et al., 2014).

Neuroinflammation is also a defining component of AD pathology. Microglia and astrocytes, the immune cells of the CNS, become chronically activated in response to Aβ plaques and damaged neurons. This persistent activation leads to the release of pro-inflammatory cytokines and chemokines, which further exacerbate neuronal damage (Bales et al., 2000). In addition, Aβ deposits in cerebral microvessels impair vascular integrity, reduce the expression of protective molecules like vascular endothelial growth factor (VEGF), and disturb blood–brain barrier function—limiting Aβ clearance and amplifying inflammation (Farkas and Luiten, 2001). Synaptic dysfunction is increasingly recognized as the primary correlate of cognitive decline in AD. Even before neuron loss becomes apparent, soluble Aβ interferes with synaptic plasticity mechanisms such as LTP, which is vital for learning and memory (Fagiani et al., 2021). Aβ also disrupts neurotransmitter systems, especially those involving glutamate, acetylcholine, and GABA (Yang et al., 2023; Ribeiro et al., 2017). For instance, overstimulation of glutamatergic pathways can lead to excitotoxicity, while loss of cholinergic neurons reduces acetylcholine availability—an observation that underpins the clinical use of cholinesterase inhibitors (Haam and Yakel, 2017). Dysregulated neurotransmission further impairs the brain’s ability to form and retain memories, highlighting the crucial link between biochemical alterations and cognitive symptoms (Handra et al., 2019). Taken together, these pathophysiological hallmarks—Aβ aggregation, tau hyperphosphorylation, oxidative stress, chronic inflammation, and synaptic failure—interact to drive the progression of AD. Understanding these interconnected mechanisms provide a foundation for developing targeted interventions aimed at modifying the course of the disease rather than just alleviating its symptoms.

## 3 Neuroprotective effects of naringenin in experimental models of neurodegeneration

Naringenin, a flavonoid abundantly found in citrus fruits, has gained considerable attention for its neuroprotective potential across various experimental models of neurodegeneration. Its mechanisms of action are broad and include antioxidant defense, anti-inflammatory effects, preservation of mitochondrial function, and modulation of neuronal signaling pathways associated with disease progression. In a cellular study (Md et al., 2018), naringenin encapsulated within nanoemulsion systems has shown enhanced protective activity against Aβ-induced toxicity. The nanoemulsion formulation improved solubility and cellular uptake, which led to significant reductions in ROS and downregulation of APP and BACE. Additionally, phosphorylation of tau protein was suppressed, indicating that naringenin interferes with key pathogenic processes in AD, including both amyloidogenesis and tauopathy (Okuyama et al., 2018). In neonatal mouse model of hypoxic–ischemic brain injury, naringenin administration resulted in reduced brain edema, oxidative stress, and neuronal apoptosis. These benefits were associated with activation of the PI3K/Akt signaling pathway and preservation of mitochondrial structure and function. Further analysis showed that VEGF might act as a molecular target, as silencing its expression diminished the protective impact of naringenin, reinforcing its role in mediating cellular resilience under hypoxic conditions (Li et al., 2024).

Neuroprotection was also evident in a rodent model of trimethyltin-induced hippocampal neurodegeneration, which mimics Alzheimer-like pathology. Treatment with naringenin restored spatial memory, improved learning performance, and significantly decreased markers of oxidative and nitrosative stress, including malondialdehyde (MDA) and nitrite (Faryadras et al., 2025). The flavonoid also reduced levels of tau phosphorylation, Aβ accumulation, and presenilin 1 expression, while preserving mitochondrial membrane potential and enhancing antioxidant enzyme activities. Histological evaluations confirmed the protection of CA1 hippocampal neurons and a marked reduction in nitrosative damage (Faryadras et al., 2025). In models investigating anesthesia-induced neurotoxicity, neonatal exposure to propofol caused widespread neuronal apoptosis and impaired adult cognitive functions. Naringenin co-treatment effectively countered these effects, leading to reduced expression of apoptotic markers such as caspase-3 and PARP, and a notable reduction in TUNEL-positive apoptotic cells. This treatment preserved normal brain morphology and maintained physiological levels of metabolic markers, such as glucose and pH, ultimately resulting in better cognitive performance in adulthood (Zou et al., 2020). Behavioral studies focusing on episodic memory demonstrated that naringenin improved both short- and long-term recognition memory in rats challenged with scopolamine. Enhanced recognition and discrimination indices were observed without affecting general locomotor activity, indicating that naringenin can support episodic memory—a form of memory often impaired in neurodegenerative conditions—without confounding effects (Ramalingayya et al., 2016; Qin et al., 2016). The compound’s neuroprotective actions were further reinforced in a model of pesticide-induced neurotoxicity. Exposure to malathion, an organophosphorus compound known to induce cholinergic dysfunction and oxidative damage, led to cognitive impairments and enzyme dysregulation. Naringenin treatment not only restored cognitive performance in object recognition and maze tasks but also normalized oxidative markers by increasing CAT and SOD activities and decreasing MDA levels. Moreover, the compound effectively inhibited AChE activity, demonstrating its potential to preserve cholinergic neurotransmission (Singh, 2024; İleritürk and Kandemir, 2023). Naringenin exhibits broad neuroprotective effects by reducing oxidative stress, mitochondrial damage, apoptosis, and amyloid/tau pathology. It also supports cholinergic function, making it a promising natural candidate for treating neurodegenerative conditions like AD and brain injury. Its low toxicity and multi-target actions justify further clinical research.

## 4 Neuroprotective mechanisms of naringenin in AD

### 4.1 Naringenin as a dual-action agent targeting oxidative stress and mitochondrial dysfunction in neuroprotection

Oxidative stress plays a critical role in the onset and progression of AD and other neurodegenerative conditions. Among the various natural compounds investigated for their neuroprotective properties, naringenin and its glycoside form naringin have shown strong potential to counteract oxidative damage and mitochondrial dysfunction, two hallmarks of neuronal degeneration (Nouri et al., 2019).

#### 4.1.1 Scavenging ROS and suppressing lipid peroxidation

In a rat model of AD-like neurodegeneration induced by intracerebroventricular streptozotocin (ICV-STZ), Khan et al. (2012) demonstrated that naringenin pretreatment (50 mg/kg, orally) markedly attenuated oxidative injury in the hippocampus. This was evident through reductions in MDA, 4-hydroxynonenal (4-HNE), thiobarbituric acid reactive substances (TBARS), and protein carbonyls, all of which are critical biomarkers of lipid and protein oxidation. Concurrently, naringenin restored the levels of glutathione (GSH), a major intracellular antioxidant, and enhanced the activity of critical detoxifying enzymes such as SOD, CAT, GPx, glutathione reductase (GR), and glutathione S-transferase. These molecular effects were associated with improved cognitive performance in passive avoidance and Morris water maze tests, as well as histological protection of hippocampal neurons, indicating a strong link between redox homeostasis and memory preservation (Khan et al., 2012). Similarly, Chtourou et al. (2014) explored naringenin’s neuroprotective effect in a rat model of iron-overload-induced cerebral toxicity—a condition known to enhance ROS production and impair mitochondrial function. Rats exposed to repeated iron dextran injections exhibited significantly elevated TBARS and protein carbonyl levels in the cerebral cortex, along with reductions in enzymatic antioxidants including SOD and CAT, and non-enzymatic molecules such as total thiols and ascorbic acid. Co-administration of naringenin effectively reversed these changes, decreasing lipid and protein oxidative markers and restoring antioxidant enzyme activities. These findings suggest that naringenin not only scavenges free radicals but also prevents iron-mediated neuronal toxicity, thereby preserving membrane integrity and cellular viability in brain tissue (Chtourou et al., 2014). Further support comes from Ma et al. (2013), who investigated the effects of naringenin on learning and memory in a rat model of AD induced by ICV-STZ. In this model, rats displayed marked cognitive deficits accompanied by oxidative damage, as indicated by increased MDA levels and reduced SOD activity in brain tissue. Naringenin administration for three weeks significantly lowered MDA content and restored SOD activity, correlating with improved escape latency and target quadrant time in the Morris water maze. Additionally, immunohistochemical analysis showed reduced expression of amyloid-beta (Aβ40 and Aβ42) in the hippocampus, and Western blotting confirmed a decrease in tau phosphorylation, both of which are downstream consequences of oxidative stress in AD pathology (Ma et al., 2013). In Ghofrani et al. (2015) study, cognitive decline was induced in rats via Aβ injection, a widely accepted method for mimicking AD-like pathology. The Aβ-injected group exhibited significant impairments in spatial learning and memory, evident through reduced performance in the Y-maze, passive avoidance, and radial arm maze tasks. These behavioral deficits were accompanied by pronounced biochemical alterations in the hippocampus, including elevated levels of MDA—a key marker of lipid peroxidation—and increased apoptosis, indicating enhanced oxidative stress. Naringenin pretreatment reduced Aβ-induced oxidative damage, as shown by significantly lower hippocampal MDA levels, indicating reduced lipid peroxidation. This suggests it neutralizes ROS or stabilizes membrane lipids, despite unchanged SOD activity. Cognitively, treated rats performed better in the Y-maze and radial arm tasks, likely due to reduced hippocampal oxidative stress. Additionally, naringenin decreased neuronal apoptosis, supporting its neuroprotective role against Aβ toxicity (Ghofrani et al., 2015).

Collectively, these studies highlight naringenin’s multifaceted antioxidant capacity. It acts both by directly neutralizing ROS and by enhancing the brain’s endogenous antioxidant defense systems. Naringenin reduces oxidative damage to lipids and proteins—two critical targets in neurodegeneration—and boosts enzymatic pathways that maintain redox balance. The reduction of MDA, 4-HNE, and TBARS, alongside the increase in GSH, SOD, and CAT, demonstrates its role in limiting lipid peroxidation and maintaining neuronal function. These actions ultimately help preserve cognitive function and structural neuronal integrity in animal models of AD, positioning naringenin as a promising neuroprotective agent against ROS-driven damage.

#### 4.1.2 Stabilizing mitochondrial function in neurodegenerative models

Loss of mitochondrial membrane potential (MMP), impaired ATP synthesis, and heightened oxidative/nitrosative stress can contribute to neuronal apoptosis and cognitive decline (Finsterer, 2012). Recent evidence has highlighted the ability of naringenin to mitigate mitochondrial deficits and restore bioenergetic balance in various experimental models of neurotoxicity. In the ICV-STZ model, which mimics sporadic AD, Sachdeva et al. (2014) demonstrated that naringin administration (50–200 mg/kg, orally) substantially ameliorated mitochondrial dysfunction and cognitive impairments. STZ-treated rats displayed a pronounced loss of MMP, accompanied by increased oxidative stress, elevated levels of pro-inflammatory cytokines (TNF-*α* and IL-1β), and reduced activities of mitochondrial enzymes critical for ATP production. In animals treated chronically with naringin, mitochondrial membrane potential was reestablished, and the activity of manganese superoxide dismutase—a key mitochondrial antioxidant enzyme essential for sustaining ATP production—was significantly restored in both the hippocampus and cortex (Sachdeva et al., 2014). This restoration was paralleled by improved performance in behavioral assays such as the Morris water maze and elevated plus maze, indicating the functional relevance of mitochondrial recovery (Sachdeva et al., 2014). Altogether, this study suggested that naringenin’s neuroprotective actions stem from its ability to mitigate mitochondrial collapse, support bioenergetic recovery, and protect against ROS-driven cellular damage. By reinforcing mitochondrial function, naringenin may play a key therapeutic role in halting or reversing the progression of neurodegenerative diseases associated with mitochondrial instability (Sachdeva et al., 2014). These protective mechanisms, confirmed across various *in vitro* and *in vivo* models, highlight naringenin’s potential as a promising therapeutic strategy to prevent or slow the progression of neurodegenerative diseases driven by oxidative damage and mitochondrial failure (Table 1).

**Table 1:** Naringenin’s antioxidant mechanisms in Alzheimer’s disease preclinical studies.

Disease/Model	Species/strain (acute/chronic)	Type of study	Intervention (route & formulation)	Key biomarkers affected	Key molecular pathways	Results (behavioral + biochemical)	Mechanistic insight	Ref
ICV-STZ-induced AD-like cognitive impairment	Wistar rat (Chronic)	*In vivo*; Behavioral + Biochemical	Naringenin 50 mg/kg, oral, 14 days prior to lesion	↑ SOD, CAT, GPx, GR; ↓ MDA, TBARS, H2O2; ↓ ChAT loss	Nrf2/ARE, Antioxidant enzymes	Improved memory (MWM); ↓ oxidative stress; ↑ enzyme levels	Nrf2 activation restored redox balance and protected neurons	Khan et al. (2012)
Aβ1-42-induced AD model	Wistar rat (Acute)	*In vivo*; Behavioral + Biochemical	Naringenin, i.p., compared with naringenin + fulvestrant	↓ MDA; ↓ apoptosis markers	ER/PI3K/Akt pathway	Improved Y-maze & RAM performance; ↓ apoptosis	Neuroprotection partially ER-mediated; ↓ lipid peroxidation	Ghofrani et al. (2015)
ICV-STZ-induced AD model	Sprague Dawley rat (Chronic)	*In vivo*; Behavioral + Biochemical	Naringenin, oral, daily for 3 weeks	↓ MDA; ↑ SOD; ↓ Aβ40/42; ↓ Tau phosphorylation	Nrf2 pathway, Tau regulation	Improved MWM performance; ↓ oxidative damage	Oxidative stress mitigation led to reduced Aβ and tau pathology	Ma et al. (2013)
ICV-STZ-induced cognitive impairment	Wistar rat (Chronic)	*In vivo*; Behavioral + Biochemical	Naringin (50, 100, 200 mg/kg), oral, 21 days	↓ MDA, NO, AChE; ↑ mitochondrial enzymes; ↓ IL-1β, TNF-α	Mitochondrial function, cytokine suppression	Improved MWM & EPM scores; ↓ oxido-nitrosative stress	Mitochondrial restoration reduced neuroinflammation	Sachdeva et al. (2014)
Iron overload-induced neurotoxicity	Wistar rat (Subacute)	*In vivo*; Biochemical + Histological	Naringenin co-administered with iron dextran (dose not specified)	↓ TBARS, protein carbonyls, NO; ↑ SOD, CAT, thiols	Oxidative stress pathways	↓ lipid peroxidation, improved antioxidant status	Antioxidant enzyme restoration prevented cortical neurotoxicity	Chtourou et al. (2014)

AD, Alzheimer’s disease; ICV-STZ-induced, Intracerebroventricular-streptozotocin-induced; SOD, Superoxide dismutase; CAT, Catalase; GPx, Glutathione peroxidase; GR, Glutathione reductase; MDA, malondialdehyde; TBARS, Thiobarbituric acid reactive substances; H₂O₂, Hydrogen peroxide; ChAT, Choline acetyltransferase; Nrf2/ARE, Nuclear factor erythroid 2–related factor 2; MWM, Morris water maze; Aβ, Amyloid-β; ER, Estrogen receptor; PI3K, Phosphatidylinositol 3-kinase; Akt, Protein kinase B; RAM, Radial Arm Maze; NO, Nitric oxide; EPM, Elevated Plus Maze; TNF-α, Tumor necrosis factor alpha; IL-1β, Interleukin-1 beta.

### 4.2 Naringenin’s modulation of amyloid and tau pathways in AD

AD is pathologically characterized by extracellular deposition of intracellular accumulation of hyperphosphorylated tau protein and Aβ plaques, both of which contribute to synaptic failure and neurodegeneration (LaFerla and Oddo, 2005). Emerging evidence from both *in vitro* and *in vivo* studies underscores the anti-amyloidogenic and tau-modulatory actions of naringenin and its synthetic analogs, mediated through direct inhibition of key enzymatic players and modulation of intracellular signaling cascades, while improvements in delivery systems have enhanced their therapeutic potential via effective BBB penetration (Table 2). A critical early event in AD pathogenesis is the aberrant processing of APP by BACE1, initiating a cascade that leads to Aβ peptide accumulation and plaque formation. In a study by Zhu et al. (2024), naringenin administration in APP/PS1 transgenic mice resulted in a significant reduction in Aβ deposition within the hippocampus and cortex. Immunohistochemical analysis confirmed decreased amyloid plaque density, while ELISA assays revealed a marked drop in soluble Aβ42 levels, supporting naringenin’s ability to modulate amyloidogenic pathways *in vivo*. Complementing this anti-amyloidogenic effect, several naringenin derivatives have been synthesized and tested for enhanced biological activity. Yang et al. (2022) reported that naringenin-O-alkylamine derivatives, particularly compounds 5f and 7 k, exhibited strong inhibitory activity against Aβ1–42 aggregation, both in its self-induced form and when catalyzed by AChE-Aβ1–40 complexes. These analogs also displayed excellent antioxidant profiles and high BBB permeability, making them suitable candidates for multifunctional AD therapy. Similarly, Mi et al. (2022) introduced naringenin-O-carbamate derivatives such as compound 3c, which showed potent inhibition of Aβ aggregation, robust neuroprotection, and the ability to disaggregate Cu^2+^-induced Aβ1–42 aggregates, a common pathway associated with metal ion dysregulation in AD brains. These findings emphasize the promise of structurally modified naringenin derivatives as multitarget-directed ligands with superior anti-amyloid capabilities.

**Table 2:** Naringenin’s anti-amyloidogenic and tau-modulatory effects.

Disease/model	Type of study	Intervention	Key biomarkers affected	Key molecular pathways	Results	Mechanistic insight	Dose dependency/ER sensitivity	Ref
APP/PS1 mice (chronic model)	*In vivo* (behavioral and biochemical)	Oral Naringenin, long-term administration	↓Aβ, ↓IL-1β, ↓TNF-α, ↓COX-2, ↓iNOS (inflammatory)	MAPK (↓p38, ↓JNK, ↓ERK1/2)	↑NORT & MWM performance, ↓Aβ plaques	MAPK inhibition attenuates gliosis and cytokine production	Yes – 100 μM most effective	Zhu et al. (2024)
Aβ25–35-induced PC12 cells (acute model)	*In vitro* (neuronal viability)	Naringenin-loaded erythrocyte membrane nanoparticles (TRNNs)	↑PSD95, ↑dendritic spine density	Nanocarrier-enhanced delivery	↑Cognition in NOR, ↑neuronal structure	Improved BBB penetration and hippocampal targeting	Not reported	Yan et al. (2024)
In silico CRMP-2 docking model	Computational	Free-form Naringenin	↓CRMP-2 phosphorylation (neurostructural)	CRMP-2 inhibition	↑Binding affinity, conformational change of CRMP-2	Prevents kinase-mediated tau phosphorylation	Not applicable	Lawal et al. (2018)
ICV-OKA Wistar rats (acute model)	*In vivo* (behavioral + biochemical)	Naringin (oral), 200 mg/kg	↓NF-κB, ↓IL-1β, ↓caspase-3, ↓AChE (inflammatory/cholinergic)	NF-κB and mitochondrial enzymes	↑MWM performance, ↓oxidative-nitrosative stress	NF-κB inhibition supports synaptic function	Yes, dose-dependent effects observed	Sachdeva and Chopra (2015)
Scopolamine-induced memory impairment (mouse model)	*In vivo* (behavioral)	Compound 5f (naringenin-O-alkylamine derivative), oral	↓AChE, ↓Aβ1-42 aggregation (cholinergic/amyloid)	Metal chelation, BBB transport	↑Memory performance, ↓aggregation	Multifunctional activity including radical scavenging and AChE inhibition	Not reported	Yang et al. (2022)
HT22 cells and *in vivo* AD model (combined)	*In vitro* + *In vivo*	Naringenin-O-carbamate derivative (compound 3c)	↓Aβ1-42 aggregation, ↓ROS, ↓iNOS (oxidative/inflammatory)	UPS activation, metal chelation	↑Protein clearance, neuroprotection	Promotes degradation of toxic aggregates via proteasome activation	Not reported	Mi et al. (2022)
Enzymatic assays + docking models	*In vitro* + computational	Naringenin carbamate derivatives (compound 1)	↓AChE, ↓BuChE, ↓Aβ aggregation (cholinergic/amyloid)	Dual-site binding at CAS/PAS, redox activity	↓Aggregation + radical scavenging	Binds both active and peripheral sites on BuChE for dual inhibition	Not reported	Wu et al. (2021)

Aβ, Amyloid-β; TNF-α, Tumor necrosis factor alpha; IL-1β, Interleukin-1 beta; MWM, Morris water maze; BBB, blood–brain barrier; MAPK, Mitogen-Activated Protein Kinase; JNK, Jun N-terminal kinase activity; ERK1/2, Extracellular signal-regulated kinases 1/2; NF-κB, Nuclear factor kappa B – A; ROS, Reactive oxygen species; AChE, acetylcholinesterase; BuChE, butyrylcholinesterase; CAS/PAS, Catalytic active site / Peripheral anionic site; NOR, Novel Object Recognition; CRMP-2, Collapsin response mediator protein 2; COX-2, Cyclooxygenase-2; UPS, Ubiquitin–proteasome system; iNOS, Inducible nitric oxide synthase.

Tau hyperphosphorylation, which destabilizes microtubules and contributes to neurofibrillary tangle formation, represents the second major molecular insult in AD. Dysregulated kinase activity, particularly by GSK-3β, is a key driver of tau pathology. Sachdeva and Chopra (2015) demonstrated that intracerebroventricular administration of okadaic acid—a known phosphatase inhibitor that mimics tauopathy—induced significant cognitive decline and tau hyperphosphorylation in rats. Treatment with naringin (the glycoside form of naringenin) substantially reversed these effects, suggesting that naringenin compounds can counteract aberrant phosphorylation events in tau-associated pathology. This modulation appears to be linked to suppression of upstream mediators like NF-κB and inflammatory cytokines, indicating that the anti-tau effect may be partially mediated via anti-inflammatory signaling. Moreover, Zhu et al. (2024) observed that naringenin alleviated neuroinflammation via suppression of the MAPK signaling cascade, a pathway known to intersect with GSK-3β and tau phosphorylation, thereby indirectly regulating tau pathology through kinase inhibition. Another promising target modulated by naringenin is collapsin response mediator protein-2 (CRMP-2), which contributes to axonal stability and tau interaction. Lawal et al. (2018) used computational modeling to reveal that naringenin binds CRMP-2 at crucial active residues, inducing conformational changes that may reduce its phosphorylation and prevent downstream tau destabilization. The authors argued that through such structural interference, naringenin could interrupt kinase-mediated CRMP-2 hyperphosphorylation—particularly by GSK-3β—thereby protecting neuronal architecture and functionality. In addition to confirming naringenin’s compatibility with CNS drug-like profiles (e.g., molecular weight, lipophilicity), this work provided a mechanistic rationale for its action at the interface of tau and axonal cytoskeletal regulation (Lawal et al., 2018).

The pharmacological efficacy of naringenin is further enhanced by the development of advanced delivery systems. Yan et al. (2024) designed biomimetic nanoparticles—TGN-decorated, erythrocyte membrane-coated PLGA nanocarriers (TRNNs)—which significantly improved BBB penetration and naringenin brain accumulation. In behavioral assays, TRNNs improved memory performance in AD mouse models, and histological assessments confirmed increased dendritic spine density and elevated PSD95 expression. *In vitro*, these nanocarriers provided robust protection to PC12 cells exposed to Aβ25-35, suggesting that nanoparticle delivery may optimize naringenin’s impact on both amyloid and tau pathologies by enhancing bioavailability and cellular uptake. Structural modifications have also played a pivotal role in enhancing target specificity and multifunctionality. Wu et al. (2021) developed naringenin-carbamate derivatives, with compound 1 displaying dual inhibition at the active and peripheral anionic sites of butyrylcholinesterase—an enzyme known to facilitate Aβ aggregation. This compound also showed superior free radical scavenging and metal-chelating activities compared to vitamin C, and its anti-Aβ aggregation activity was validated through *in vitro* assays. These multifunctional profiles position such derivatives as promising candidates for broad-spectrum intervention across amyloid, tau, and oxidative pathways. Taken together, the cumulative evidence across these studies delineates a multifaceted role for naringenin in counteracting key molecular events in AD. Through direct inhibition of BACE1and suppression of APP processing, naringenin reduces Aβ synthesis and deposition. Its impact on tau pathology is mediated through interference with GSK-3β and MAPK signaling, downregulation of total and phosphorylated tau, and interaction with structural proteins like CRMP-2. Moreover, its efficacy is magnified by rational structural modifications and advanced BBB-penetrant nanocarriers, which overcome pharmacokinetic limitations (Wu et al., 2021). These collective findings not only establish naringenin and its analogs as potent anti-amyloid and anti-tau agents, but also highlight the therapeutic promise of multifunctional, bioavailable compounds in tackling the complex etiology of AD.

### 4.3 Anti-inflammatory mechanisms of naringenin in AD

**Figure 1:**
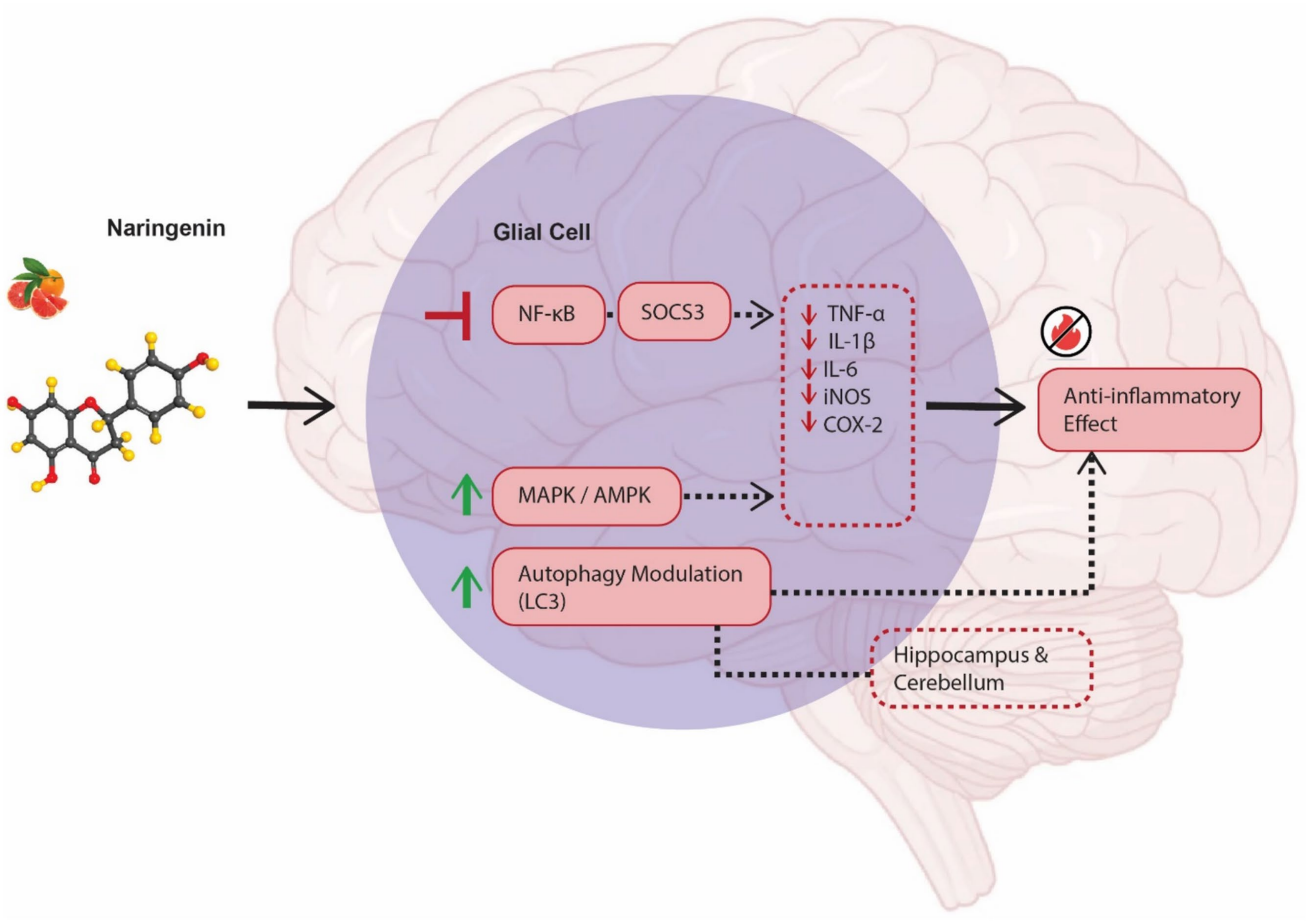
Anti-inflammatory mechanisms of naringenin in Alzheimer’s disease (AD). This figure illustrates the multi-target anti-inflammatory effects of naringenin (NRG) in the context of AD pathology. Naringenin modulates key inflammatory signaling pathways—including nuclear factor kappa B (NF-κB), mitogen-activated protein kinase (MAPK), suppressor of cytokine signaling 3 (SOCS3), and AMP-activated protein kinase (AMPK)—leading to the suppression of pro-inflammatory mediators such as interleukin-1β (IL-1β), interleukin-6 (IL-6), tumor necrosis factor-alpha (TNF-*α*), inducible nitric oxide synthase (iNOS), and cyclooxygenase-2 (COX-2). These molecular changes result in reduced glial activation, decreased cytokine expression, and attenuated oxidative stress. Experimental models of AD further confirm that naringenin and its glycoside naringin reduce iNOS and tau overexpression, restore glutathione (GSH) levels, downregulate lipid peroxidation, and improve spatial learning and coordination. Additionally, naringenin modulates autophagy-related markers such as LC3, indicating its role in neuroinflammatory control and neuronal protection across multiple brain regions including the hippocampus and cerebellum.

Naringenin has gained considerable attention for its potent anti-inflammatory activities, particularly in neurodegenerative diseases like AD (Figure 1). Several studies have demonstrated its ability to inhibit major inflammatory mediators such as IL-6, TNF-*α*, IL-1β, inducible nitric oxide synthase (iNOS), and cyclooxygenase-2 (COX-2), as well as to regulate key intracellular signaling pathways, including NF-κB and MAPK. Importantly, recent findings also highlight the involvement of suppressor of cytokine signaling 3 and AMP-activated protein kinase (AMPK) in mediating its protective actions (Zhang et al., 2023; Umukoro et al., 2018; Ali et al., 2017). In a recent experimental investigation, Hassan et al. (2022) explored the neuroprotective effects of naringin in a rat model of aluminum chloride (AlCl₃)-induced AD. Their study revealed that exposure to AlCl₃ led to notable cerebellar dysfunction, oxidative imbalance, and behavioral deficits. Rats subjected to AlCl₃ exhibited impaired spatial learning and motor coordination, alongside elevated lipid peroxidation and a reduction in endogenous antioxidant reserves, particularly GSH. Immunohistochemical analysis of cerebellar tissue further demonstrated an upregulation of pro-inflammatory markers, including inducible iNOS, and elevated tau expression, indicating ongoing oxidative and inflammatory injury. Administration of naringin (100 mg/kg/day, orally) for 21 days substantially attenuated these alterations. Naringin significantly suppressed lipid peroxidation and restored GSH levels in both the hippocampus and cerebellum. Moreover, it downregulated iNOS expression in cerebellar tissue, highlighting its anti-inflammatory efficacy (Hassan et al., 2022). The compound also normalized autophagy markers such as LC3 and reduced pathological tau accumulation, suggesting that its neuroprotective effects extend beyond antioxidant activity to include modulation of neuroinflammatory and autophagic responses. These findings position naringin as a multi-target agent capable of protecting cerebellar structure and function by dampening both oxidative and inflammatory mechanisms (Hassan et al., 2022). Zhu et al. (2024) investigated the anti-inflammatory potential of naringenin in AD, emphasizing its ability to reduce glial activation and cytokine production. In APP/PS1 transgenic mice, chronic administration of NRG significantly reduced the number of reactive microglia and astrocytes near Aβ plaques, as confirmed by immunohistochemical and molecular analyses. Naringenin treatment also led to a marked decline in the expression of pro-inflammatory markers such as TNF-*α* and IL-1β in brain regions commonly affected in AD.

### 4.4 Cholinergic system modulation by naringenin: inhibition of cholinesterases and enhancement of cognitive function

Modulation of the cholinergic system remains a core therapeutic target in AD, primarily due to the progressive loss of cholinergic neurons and the decline in acetylcholine (ACh) levels that characterize the disease (Hampel et al., 2018). Naringenin and its derivatives have demonstrated promising effects on this system by acting as natural inhibitors of cholinesterase enzymes and enhancing cognitive performance across various models of neurodegeneration (Heo et al., 2004; Kumar et al., 2010). The cholinergic system plays a fundamental role in regulating memory and cognitive functions, particularly in the context of neurodegenerative diseases such as AD. One of the principal pathological hallmarks of AD is the reduction of ACh levels, often due to the overactivity of cholinesterase enzymes—AChE and BuChE. Inhibiting these enzymes has been a central pharmacological strategy for mitigating cognitive decline. Recent research highlights the potential of naringenin, a citrus-derived flavonoid, in modulating cholinergic signaling by acting as a natural inhibitor of AChE and BuChE and restoring cholinergic neurotransmission. In a recent study, Wu et al. (2021) developed naringenin carbamate derivatives to address cholinergic dysfunction in AD. These compounds inhibited both AChE and BuChE, with compound 1 showing potent BuChE inhibition. Molecular docking confirmed dual-site targeting, enhancing synaptic acetylcholine levels and supporting cholinergic signaling. This modulation could improve cognitive deficits. Additionally, the compounds showed antioxidant and anti-amyloid properties, but their primary therapeutic value lies in restoring cholinergic balance through effective cholinesterase inhibition (Wu et al., 2021). In a study by Haider et al. (2020), the neuroprotective effects of naringenin were explored using a rat model of AD induced by aluminum chloride (AlCl₃) and D-galactose. Their findings revealed that oral pre-treatment with naringenin (50 mg/kg) significantly reduced AChE activity in both the hippocampus and cortex—two brain regions critically involved in learning and memory. Notably, this suppression of AChE activity was comparable to the effects observed with donepezil, a standard cholinesterase inhibitor. Furthermore, gene expression analysis demonstrated that naringenin effectively downregulated AChE mRNA levels, suggesting transcriptional regulation as one of its mechanisms of action.

Alongside its inhibitory action on AChE, naringenin also appears to modulate downstream cholinergic functions. The same study by Haider et al. (2020) reported improvements in behavioral performance following naringenin treatment, including better memory retention and reduced anxiety-like behavior in treated animals. These cognitive enhancements correlated with a restoration of acetylcholine availability in synaptic clefts, thereby supporting its role in maintaining cholinergic tone. This neuromodulatory effect of naringenin was also associated with increased activity of dopaminergic and serotonergic pathways, indicating a broader spectrum of neurotransmitter system involvement. Complementing these findings, Lai et al. (2022) investigated the synergistic effects of naringenin with rivastigmine, a clinically approved cholinesterase inhibitor, in an AlCl₃-induced dementia model. In their experiment, rats receiving a combination of subtherapeutic rivastigmine (1.25 mg/kg) and naringenin (100 mg/kg) demonstrated significantly greater improvements in behavioral and biochemical outcomes compared to either agent alone. The co-administration of these compounds resulted in more effective inhibition of both AChE and BuChE, two key enzymes responsible for acetylcholine degradation. Not only did this dual inhibition boost synaptic acetylcholine levels, but it also yielded superior protection against aluminum-induced cognitive deficits (Lai et al., 2022). The influence of naringenin on the cholinergic system was further supported by *in vitro* investigations using SK-N-AS neuroblastoma cells, as reported by Kuşi et al. (2023). In this cellular model of AD induced by Aβ₍₂₅_−_₃₅₎, treatment with naringenin led to a marked reduction in Aβ accumulation and associated neurotoxicity. Although this study primarily focused on neuroprotection, it indirectly supported the involvement of cholinergic modulation by demonstrating reduced cellular stress and improved neuronal viability—key outcomes often associated with elevated acetylcholine signaling. Interestingly, naringin—closely related to naringenin—also exerted similar protective effects, though naringenin appeared more potent in reducing amyloidogenic and tau-related toxicity. Another critical aspect of naringenin’s cholinergic modulation lies in its action on BuChE (Kuşi et al., 2023). While AChE primarily hydrolyzes acetylcholine under physiological conditions, BuChE plays an increasing role in AD progression, especially when AChE activity declines in advanced disease stages. The enhanced inhibition of BuChE by naringenin, as observed in Lai et al. (2022), suggests its potential utility even in later phases of cognitive impairment, offering sustained cholinergic support where traditional AChE inhibitors may falter.

In addition to enzymatic inhibition, naringenin may also influence the structural integrity of cholinergic neurons. Studies have noted its antioxidant properties, which protect neuronal membranes and synaptic structures from oxidative damage—a common driver of cholinergic neuron degeneration. By reducing lipid peroxidation and enhancing antioxidant defenses, naringenin helps preserve the functional integrity of cholinergic circuits, creating a more favorable environment for neurotransmission and plasticity (Lai et al., 2022). Taken together, these studies converge on the conclusion that naringenin exerts multifaceted modulation of the cholinergic system through inhibition of AChE and BuChE, upregulation of acetylcholine levels, and protection of neural substrates. Importantly, its ability to synergize with existing drugs like rivastigmine opens new avenues for combination therapies that may require lower doses of synthetic cholinesterase inhibitors, potentially reducing their side effects. Moreover, its capacity to influence gene expression and oxidative pathways underscores its potential as a disease-modifying agent, rather than merely a symptomatic treatment.

### 4.5 Modulation of synaptic plasticity and neurotrophic pathways

Synaptic plasticity, the ability of synapses to strengthen or weaken over time, is a crucial process underlying memory and learning (Basu and Siegelbaum, 2015). Naringin, a bioactive flavonoid predominantly found in citrus fruits, has shown potential to improve synaptic function and memory through multiple neurotrophic signaling pathways. Recent evidence highlights its ability to enhance hippocampal LTP, a well-known marker of synaptic strength, and to modulate critical molecular cascades such as BDNF–TrkB-CREB (Alijani et al., 2025). Meng et al. (2021) demonstrated that naringin markedly improves memory performance in hydrocortisone-induced cognitive impairment in mice by targeting various molecular systems. Behavioral tests such as the Morris water maze and novel object recognition revealed significant improvements in spatial and recognition memory following naringin administration. At the molecular level, naringin enhanced the expression of ERα and ERβ in the hippocampus, as shown through immunohistochemistry. This upregulation was associated with downstream engagement of the MAPK/P38 signaling cascade, which plays a key role in regulating synaptic plasticity and neuronal survival. These findings suggest that naringin mediates neuroprotection not only through anti-amyloid and anti-apoptotic actions but also by restoring synaptic architecture and function via estrogen receptor signaling and MAPK pathway engagement (Meng et al., 2021). Further extending these observations, Choi et al. (2023) explored the electrophysiological correlates of naringin treatment in an A*β*-induced AD rat model. Using organotypic hippocampal slice cultures, they found that naringin significantly enhanced LTP, a cellular correlate of learning and memory. Naringin administration reversed the Aβ-induced suppression of field excitatory postsynaptic potentials, especially within the CA1 region of the hippocampus, highlighting its synaptic restorative effect. The compound improved behavioral performance in spatial and recognition memory tasks and significantly modulated key proteins within neurotrophic signaling cascades. Specifically, naringin increased the expression of BDNF, its receptor TrkB, and phosphorylated CREB, while simultaneously reducing COX-2 and Bax expression (Choi et al., 2023). These findings underscore the therapeutic relevance of BDNF–TrkB-CREB signaling in counteracting the deleterious synaptic effects of Aβ pathology, with naringin as a potent modulator of this axis.

The enhancement of memory and synaptic integrity by naringin has also been linked to the activation of calcium/calmodulin-dependent protein kinase II (CaMKII). Wang et al. (2013) evaluated this in APPswe/PS1dE9 transgenic mice, a well-established AD model. Their findings revealed that chronic treatment with naringin (100 mg/kg/day for 16 weeks) significantly increased phosphorylation at the Thr286 site of CaMKII, converting the enzyme to its active form. This autophosphorylation is essential for synaptic strengthening and long-term memory formation. In addition, naringin upregulated the phosphorylation of AMPA receptors at CaMKII-dependent sites, facilitating excitatory synaptic transmission. Behavioral assessments confirmed improved cognitive outcomes, particularly in long-term spatial memory, in treated animals. Notably, these effects exceeded those achieved by the reference drug Aricept, which did not enhance CaMKII activity. The results implicate CaMKII as a central effector in naringin’s action and suggest that its activation represents a key therapeutic strategy for rescuing synaptic loss in AD (Wang et al., 2013). In addition to its direct effects on molecular signaling, naringin also supports neuroplasticity through interaction with metabolic and redox pathways. Salehpour et al. (2023) investigated the combined effects of naringin supplementation and aerobic exercise on Aβ-induced AD model rats. The combination therapy significantly improved spatial learning and memory, as assessed by the Morris water maze. Molecular analysis revealed upregulation of hydrogen sulfide (H₂S) pathway markers, including increased levels of cystathionine β-synthase (CBS) and S-adenosylmethionine (SAM), suggesting enhanced endogenous H₂S production. H₂S is increasingly recognized for its neuroprotective roles, particularly in promoting synaptic integrity and reducing neuronal loss. The beneficial effects observed with naringin, especially when paired with exercise, may thus arise from an integrated action on synaptic signaling and neurometabolic health (Salehpour et al., 2023).

Altogether, these findings from four independent studies provide compelling evidence that naringin exerts its neuroprotective effects by modulating key pathways governing synaptic plasticity. Through ERα/ERβ stimulation, naringin activates the MAPK signaling pathway, which facilitates synaptic maintenance and anti-apoptotic processes (Meng et al., 2021). Its positive modulation of LTP, mediated via BDNF–TrkB-CREB signaling, underlies its ability to rescue memory deficits in Aβ-challenged brains (Choi et al., 2023). In parallel, the activation of CaMKII by naringin restores phosphorylation dynamics critical for LTP and memory retention (Wang et al., 2013). Furthermore, the concurrent enhancement of H₂S signaling points to a broader metabolic and redox regulatory mechanism, especially when combined with physical activity (Salehpour et al., 2023). The convergence of these signaling pathways suggests that naringin does not act via a single mechanism but instead orchestrates a network of neuroprotective responses. This multifaceted action positions naringin as a promising candidate for slowing or reversing synaptic dysfunction in AD. Given its favorable safety profile and bioavailability, particularly in the CNS, naringin holds translational potential for integration into early-stage AD management or as an adjunct to existing therapies. In conclusion, naringin enhances synaptic signaling and cognitive recovery by activating estrogen receptor-mediated and MAPK-dependent pathways, stimulating BDNF–TrkB-CREB signaling, promoting CaMKII activation, and boosting H₂S production. These synergistic effects culminate in improved LTP and preservation of hippocampal synaptic integrity, thereby offering a scientifically supported, multi-targeted approach to AD intervention (Table 3).

**Table 3:** Naringenin-mediated modulation of synaptic plasticity in Alzheimer’s disease models.

Disease/model	Type of study	Intervention	Key biomarkers affected	Key molecular pathways	Results	Mechanistic insight	Ref
Hydrocortisone-induced memory impairment in ICR mice (acute)	*In vivo* (behavioral + biochemical)	Naringin (oral, 100 mg/kg/day), ER antagonist group included	Oxidative: SOD↑, MDA↓, NO↓; Inflammatory: IL-1β↓; Apoptotic: caspase-3↓, Bcl-2↑; Cholinergic: Ach↑, AchE↓	ER/PI3K/Akt, MAPK/p38, Cholinergic, Glutamatergic	Improved MWM + NOR performance; restored hippocampal ultrastructure	ER-dependent modulation of multiple AD pathways leads to synaptic protection	Meng et al. (2021)
Aβ-injected Sprague–Dawley rats (acute)	*In vivo* + *ex vivo* (electrophysiology + behavioral)	Naringin (oral, 100 mg/kg/day for 20 days)	Inflammatory: COX-2↓; Apoptotic: Bax↓, Bcl-2↑; Neurotrophic: CREB↑, BDNF↑, TrkB↑	BDNF/TrkB/CREB pathway	Rescued LTP in CA1; improved MWM, NOR, and passive avoidance	Neurotrophic support via BDNF axis improves synaptic strength and plasticity	Choi et al. (2023)
APPswe/PS1dE9 transgenic mice (chronic)	*In vivo* (behavioral + biochemical)	Naringin (oral, 50 or 100 mg/kg/day for 16 weeks)	Synaptic plasticity: CaMKII↑, AMPAR-p↑; Apoptotic: GSK-3β↓	CaMKII/GSK-3β signaling axis	Improved long-term memory in MWM; synaptic protein expression restored	Enhanced CaMKII activity restores synaptic function and memory	Wang et al. (2013)
Aβ-injected Wistar rats (acute)	*In vivo* (behavioral + biochemical)	Naringin (oral, 80 mg/kg/day for 4 weeks) with or without aerobic training	H2S signaling: SAM↑, CBS↑, H2S↑; Neuronal loss ↓	H2S signaling pathway	Improved MWM; elevated CBS and H2S; reduced hippocampal cell death	H2S pathway activation prevents cell death and enhances cognition	Salehpour et al. (2023)

AD, Alzheimer’s disease; SOD, Superoxide dismutase; MDA, malondialdehyde; NO, Nitric oxide; IL-1β, Interleukin-1 beta; PI3K, Phosphatidylinositol 3-kinase; Akt, Protein kinase B; MWM, Morris water maze; ER, Estrogen receptor; CaMKII, Calcium/Calmodulin-dependent Protein Kinase II; GSK-3β, Glycogen Synthase Kinase 3 Beta; AMPAR-p, α-amino-3-hydroxy-5-methyl-4-isoxazolepropionic acid receptors; CREB, cAMP Response Element-Binding Protein; TrkB, Tropomyosin receptor kinase B; COX-2, Cyclooxygenase-2; SAM, S-adenosylmethionine upregulation; CBS, Cystathionine-β-synthase; H₂S, Hydrogen sulfide; APPswe/PS1dE9, Amyloid Precursor Protein (Swedish mutation) and Presenilin-1 deletion; MAPK/p38, Mitogen-Activated Protein Kinase / p38.

### 4.6 Epigenetic and hormonal regulation and the gut–brain axis in naringin-mediated neuroprotection

Recent findings underscore the multifaceted therapeutic potential of naringin, a bioactive dihydroflavonoid, in modulating neurodegeneration through epigenetic remodeling, estrogen receptor signaling, and microbiota-mediated gut–brain interactions. These interconnected mechanisms offer a broader understanding of how naringin could impact the pathophysiology of AD at both systemic and cellular levels.

#### 4.6.1 Epigenetic remodeling and insulin signaling pathways

One of the hallmark features of AD involves dysregulation in cerebral insulin signaling and energy metabolism. Yang et al. (2014) investigated the neuroprotective effects of naringenin (the aglycone form of naringin) in a STZ-induced AD model in rats. In animal model, naringenin has been reported to elevate mRNA expression of insulin and its receptor in both the hippocampus and cortex, suggesting enhanced central insulin signaling. Although such direct transcriptional modulation in human brain tissue has not yet been confirmed, related studies in human liver cells indicate that naringenin downregulates IRE1α expression, alleviates ER stress, and restores insulin responsiveness, pointing to its regulatory effects on insulin signaling at the gene expression level in human systems (Jia et al., 2019).

This modulation was accompanied by decreased tau hyperphosphorylation via downregulation of GSK-3β, a kinase heavily implicated in AD-related tau pathology (Yang et al., 2014). The downstream molecular adaptations suggest that naringenin may exert epigenetic control over insulin-associated gene networks. Although the precise epigenetic mechanisms were not delineated, the increased expression of insulin-degrading enzyme observed in the study indicates a potential influence on transcriptional regulation. Given that hyperphosphorylation of tau and impaired insulin signaling are epigenetically linked through chromatin remodeling and histone acetylation, it is plausible that naringenin contributes to DNA methylation balance and histone modification patterns in key neurogenic loci. Moreover, such epigenetic modulation likely enhances neuronal resilience and synaptic maintenance, particularly under metabolic stress (Yang et al., 2014).

#### 4.6.2 Hormonal regulation through estrogen receptor activation

Naringin’s interaction with estrogen receptor pathways provides a second layer of neuroprotection, particularly through ERα and ERβ signaling. As highlighted by Wang et al. (2012), long-term naringin supplementation in the APPswe/PSΔE9 mouse model of AD improved glucose uptake and synaptic function. This effect was partially attributed to increased phosphorylation of GSK-3β and activation of PI3K/Akt signaling—a canonical downstream cascade of estrogen receptors.

Although ER-dependent transcriptional control was not the primary focus of this study, the observed neuroprotective effects are consistent with known estrogen-mediated activation of PI3K/Akt and CREB pathways. ERα and ERβ are nuclear receptors that, upon ligand binding, modulate gene expression through direct interaction with estrogen response elements (EREs) or via cross-talk with transcription factors such as FOXO and CREB (Wang et al., 2012). Naringin’s partial mimicry of estrogen, especially in restoring PI3K/Akt activity and reducing neurotoxicity, supports its functional role as a phytoestrogen. Ghofrani et al. (2015) demonstrated that naringenin significantly improves cognitive performance in an AD rat model, with effects strongly linked to ER pathways. Co-treatment with fulvestrant, an ER antagonist, abolished naringenin’s benefits, confirming its reliance on ERα and ERβ. These findings suggest that naringenin may function as a selective estrogen receptor modulator, triggering downstream neuroprotective mechanisms. The compound also reduced lipid peroxidation and apoptosis in the hippocampus (Ghofrani et al., 2015). Furthermore, previous studies have suggested that flavonoids like naringin can influence the recruitment of coactivators or repressors to estrogen receptors, leading to selective gene activation. Thus, the modulation of GSK-3β and insulin pathways observed in both (Yang et al., 2014; Wang et al., 2012) may arise not only from direct kinase inhibition but also from estrogen receptor-mediated epigenetic reprogramming.

#### 4.6.3 Gut–brain axis and microbiota modulation

Beyond the CNS, naringin demonstrates promising activity in modulating gut microbiota, which in turn may impact brain function via the gut–brain axis. Zhu et al. (2020) reported that naringin significantly extended the lifespan of *C. elegans* and delayed aging-related phenotypes in both AD and Parkinson’s disease models. Notably, these effects were dependent on DAF-16, a FOXO transcription factor regulated by the insulin/IGF-1 signaling (IIS) pathway, suggesting systemic epigenetic control across tissues. While direct microbiota composition was not measured in the *C. elegans* model, previous research has established a close link between FOXO signaling and microbial diversity. Naringin’s upregulation of daf-16-regulated genes may facilitate enhanced host–microbe interactions, thereby boosting metabolic and immunological defenses against neurodegeneration (Zhu et al., 2020). Moreover, gut-derived metabolites, such as SCFAs, are key mediators of the gut–brain axis, capable of modulating blood–brain barrier permeability, neuroinflammation, and even histone acetylation in neural tissue. Although SCFAs were not directly quantified in the aforementioned studies, naringin’s role in enhancing energy metabolism and decreasing oxidative stress implies an indirect boost to beneficial SCFA-producing microbes. These microbial metabolites could participate in CNS anti-inflammatory signaling and chromatin remodeling, ultimately enhancing brain homeostasis. Through this pathway, naringin may function as a dietary modulator of the gut microbiota, promoting an anti-inflammatory milieu that supports cognitive health (Zhu et al., 2020).

#### 4.6.4 Systemic-to-CNS communication in AD

The convergence of hormonal, metabolic, and microbial signaling pathways illustrates the importance of systemic-to-CNS communication in AD pathogenesis and its modulation by naringin. The restoration of insulin signaling in the brain, as shown by Yang et al. (2014), not only improves neuronal glucose uptake but also intersects with PI3K/Akt-dependent neurotrophic signaling, crucial for synaptic plasticity and memory encoding. Furthermore, Wang et al. (2012) showed that naringin increased locomotor activity and reduced senile plaque density in transgenic mice, consistent with improved CNS integration of systemic metabolic cues. These effects might also involve changes in microglial activation states or cytokine production, although these specific markers were not explored in detail. On a systemic level, Zhu et al. (2020) provided evidence for naringin’s ability to delay disease progression in aging models by promoting longevity-associated gene expression. This suggests a potential epigenetic mechanism bridging peripheral and central aging pathways via conserved signaling intermediates such as DAF-16/FOXO and sirtuins, which are also influenced by estrogen signaling and nutrient-sensing pathways (Zhu et al., 2020). Together, these findings reveal that naringin, via its influence on epigenetic regulation, estrogen receptor signaling, and the gut–brain axis, holds promise as a multi-target agent in AD therapy. It modulates brain insulin sensitivity, suppresses tau pathology, interacts with ERα/ERβ to trigger neuroprotective PI3K/Akt cascades, and potentially reshapes gut microbiota to produce beneficial SCFAs. These converging mechanisms form a coherent framework for systemic-to-CNS neuroprotection, positioning naringin as a candidate for further translational research in aging and neurodegenerative disease management.

## 5 Nanoformulations and drug delivery strategies for naringenin in neurodegenerative disorders

The advancement of naringenin from preclinical promise to clinical application in AD therapy depends heavily on its pharmacokinetic characteristics, ability to cross the BBB, safety profile, and mechanistic action across relevant neural pathways. Naringenin is a hydrophobic flavonoid with poor aqueous solubility, a property that limits its systemic absorption and oral bioavailability (Shulman et al., 2011). While this hydrophobic nature may favor its interaction with lipid-rich biological membranes such as the blood–brain barrier, efficient BBB penetration often still requires carrier systems. As reported in recent studies, including micelle-based delivery systems, hydrophobic interactions can indeed be exploited to facilitate brain targeting (Wu et al., 2023). Therefore, although naringenin’s low water solubility can support BBB permeability in theory, its poor pharmacokinetic profile necessitates formulation strategies such as nanoparticles or liposomes to ensure effective delivery to the central nervous system. To overcome these pharmacokinetic challenges, recent advances have introduced a range of nanoparticle-based delivery systems that significantly enhance its brain penetration, stability, and sustained release (Ji et al., 2016; Li et al., 2017). In a neonatal model of anesthetic-induced neurodegeneration, mice exposed to propofol, which caused marked apoptotic neuronal loss and metabolic disturbance, co-administration of naringenin (50 mg/kg) restored neuronal integrity and improved adult cognitive performance. Gene expression analyses confirmed the downregulation of pro-apoptotic markers such as caspase-3 and PARP following naringenin treatment, suggesting strong anti-apoptotic potential. These findings support that early-life exposure to naringenin may impart neurodevelopmental resilience that persists into adulthood (Zou et al., 2020). However, achieving effective concentrations of naringenin in the CNS remains a key hurdle, largely due to the BBB. The docking simulations showed high binding affinities of naringenin to acetylcholinesterase and butyrylcholinesterase, both implicated in AD progression. Additionally, moist heat-treated laurel extracts enriched with naringenin exhibited superior antioxidant and anticholinesterase activity, supporting the functional viability of naringenin as a CNS-active molecule (Al-Rajhi et al., 2023).

To translate these bioactivities into viable therapies, nanoformulation techniques have been developed to facilitate brain-specific delivery (Sarvaiya and Agrawal, 2015). Chitosan-based naringenin nanoparticles (NAR-CNPs) using ionic gelation for direct nose-to-brain transport (Chakraborty et al., 2025). The nanoparticles had a mean size of approximately 112 nm with favorable zeta potential and demonstrated sustained drug release over eight hours. Permeability studies confirmed enhanced transnasal flux compared to non-encapsulated forms. In a murine model of scopolamine-induced cognitive impairment, NAR-CNPs not only improved memory and behavioral outcomes but also modulated brain oxidative stress by lowering malondialdehyde levels and enhancing enzymatic antioxidants. These outcomes underscore the promise of intranasal chitosan nanoparticles in bypassing the BBB for neurodegenerative therapy (Chakraborty et al., 2025). In a more advanced strategy, brain-targeted nanoparticles were developed by modifying poly(ethylene glycol)-poly(*ε*-caprolactone) (PEG-PCL) carriers with Angiopep-2, a peptide known for its strong ability to penetrate the BBB (Rabha et al., 2021). These Angiopep-2-functionalized naringin-loaded nanoparticles (ANG-NG-NPs) exhibited favorable physicochemical characteristics, including a particle size around 126 nm and an encapsulation efficiency of approximately 73%. *In vivo* studies demonstrated that this delivery system significantly enhanced brain accumulation of naringin and improved cognitive performance in APP/PS1 transgenic mice. Furthermore, hippocampal examination revealed a reduction in hyperphosphorylated tau protein levels, indicating a potential for modifying disease progression. The extended circulation time and sustained release profile further underscore the suitability of this platform for targeted CNS therapies (Jiang et al., 2025; Pecorini et al., 2023; Rakotondrabe et al., 2023). In terms of therapeutic targeting, naringenin and its derivatives have also been studied for their ability to inhibit AChE, a key enzyme involved in cholinergic neurotransmission. Several synthetic analogs demonstrated improved inhibitory effects compared to naringenin itself, as validated through enzymatic assays and molecular docking. These derivatives interacted strongly with catalytic and peripheral residues of the AChE active site, offering structural leads for future drug development (Tran et al., 2021).

Finally, behavioral studies in ICV-STZ models confirmed that oral administration of naringenin (50 mg/kg/day) for 3 weeks significantly improved cognitive function in rats, as evidenced by better memory retention in maze-based tasks. These *in vivo* outcomes not only validate its efficacy but also provide foundational dosing insights for translational studies (Baluchnejadmojarad and Roghani, 2006). These studies collectively reveal a paradigm shift in the delivery of flavonoid-based therapeutics like naringenin for neurodegeneration. Traditional systemic administration is severely limited by the molecule’s physicochemical properties, while nanoformulations—especially those using chitosan, PEG-PCL, or emulsifying systems—demonstrate a reliable means of improving delivery efficiency, bioavailability, and clinical impact. Moreover, biomimetic strategies such as Angiopep-2 functionalization provide added specificity and permeability to traverse the BBB, addressing one of the most significant barriers in CNS drug delivery (Pecorini et al., 2023; Xin et al., 2011). However, future research must involve rigorous pharmacokinetic profiling, long-term safety assessments, and human clinical trials to confirm its therapeutic viability.

### 5.1 Nano-based naringin therapy in AD

A particularly innovative approach involved the creation of a naringenin delivery system utilizing erythrocyte membrane-coated poly(lactic-co-glycolic acid) nanoparticles modified with the brain-targeting TGN peptide. This delivery platform, termed TRNNs, exhibited markedly enhanced permeability in an *in vitro* co-culture BBB model—achieving over a seven-fold increase in transport efficiency compared to unmodified nanoparticles (Na et al., 2023). *In vivo* imaging confirmed high brain accumulation of these particles, nearly eight times greater than conventional systems. Behavioral tests further demonstrated that TRNNs improved recognition memory in AD model mice. These cognitive benefits were supported at the molecular level by increased hippocampal PSD95 expression and enhanced dendritic spine density, indicating structural and synaptic restoration in affected neurons. Additionally, *in vitro* assays using A*β*25-35-injured PC12 cells revealed significant neuroprotection following TRNN pre-treatment, confirming the ability of this functionalized system to mitigate Aβ-induced cytotoxicity (Yan et al., 2024; Zeng et al., 2023). Parallel to injectable nanocarriers, oral formulations have also demonstrated therapeutic viability. In another study, naringin was incorporated into tablet forms using micro/nanocrystalline cellulose derived from agro-waste residues. These solid oral tablets were evaluated in an AD rat model induced by D-galactose and AlCl₃ administration. The naringin tablets significantly improved cognitive and spatial memory performance, reduced oxidative stress, normalized neurotransmitter levels, and suppressed neuroinflammation (Helmy et al., 2023). Histological analyses revealed restored cortical and hippocampal architecture. Interestingly, both micro- and nanocrystalline cellulose-based naringin tablets outperformed the standard reference drug donepezil in reversing neurodegenerative pathology and behavioral deficits, highlighting their superior neuroprotective potential (Helmy et al., 2023). Together, these findings demonstrate that both injectable biomimetic nanocarriers and advanced oral tablet technologies are viable options for improving the delivery and efficacy of citrus flavonoids in AD. By enhancing brain bioavailability and preserving neuronal integrity, such formulations represent promising avenues for future neurotherapeutic development.

## 6 Combined interventions

### 6.1 Synergistic effect of naringenin and exercise in AD models

Recent findings emphasize the therapeutic potential of combining aerobic exercise with naringenin supplementation in mitigating AD-associated cognitive decline. Dibaee et al. (2021) conducted a study to investigate how this combination influences memory function and hippocampal adiponectin levels in a rat model of AD induced by Aβ₁_−_₄₂ injection. Their results demonstrated that both aerobic training and naringenin (80 mg/kg/day) independently enhanced spatial learning and memory, but the most significant improvements were observed when both interventions were applied together. Over a four-week period, rats subjected to combined aerobic exercise and naringenin treatment (ADETN group) showed superior performance in behavioral memory tests and elevated hippocampal adiponectin levels compared to either treatment alone (ADET or ADN groups). This suggests a synergistic mechanism, where exercise-induced metabolic regulation and naringenin’s neuroprotective effects jointly modulate hippocampal function (Dibaee et al., 2021). Adiponectin, a key adipokine linked to synaptic plasticity and insulin signaling in the brain, appears to mediate these benefits (Formolo et al., 2022). Increased adiponectin expression following the combined treatment may facilitate neuronal resilience against amyloid-induced toxicity, potentially slowing AD progression. Salehpour et al. (2023) demonstrated that combining naringin with treadmill exercise significantly improved spatial memory in Aβ-injected rats. This cognitive enhancement was linked to elevated hippocampal levels of hydrogen sulfide (H₂S), SAM, and cystathionine-β-synthase (CBS). As H₂S functions as both a neuromodulator and antioxidant, its increase likely supports neuronal survival (Salehpour et al., 2023). Together, these findings suggest that the concurrent use of naringenin or naringin with aerobic exercise promotes superior neurocognitive benefits compared to either approach alone, through mechanisms involving both adiponectin signaling and H₂S-mediated neuroprotection. This combination strategy holds promise for non-pharmacological intervention in neurodegenerative diseases like AD.

### 6.2 Naringenin improves cognition in aging via multifactorial neuroprotection

In a recent study by Zhou et al. (2020) the neuroprotective properties of naringenin were evaluated in high-fat diet-fed senescence-accelerated mouse prone 8 (SAMP8) mice, a model mimicking age-related Alzheimer’s pathology. Daily dietary supplementation with 0.2% naringenin over 12 weeks led to marked improvements in spatial learning and memory, as demonstrated by the Barnes Maze and Morris Water Maze tasks. Mechanistic investigations revealed that naringenin exerted its effects by attenuating key pathological features of AD (Zhou et al., 2020). These included reduced Aβ accumulation, decreased tau hyperphosphorylation, and mitigation of oxidative stress and neuroinflammatory responses in the brain. The findings suggest that naringenin’s cognitive benefits stem from its ability to modulate multiple degenerative pathways simultaneously. This work reinforces the therapeutic potential of naringenin as a dietary intervention in age-associated cognitive decline, particularly in metabolic or inflammation-exacerbated models of neurodegeneration (Zhou et al., 2020). Such outcomes highlight its promise for future AD treatment strategies targeting multifaceted disease mechanisms.

## 7 Conclusion

Naringenin has emerged as a promising multi-functional agent in AD research, owing to its ability to target several core pathological processes. One of the most critical mechanisms underlying its neuroprotective action is the activation of the Nrf2/ARE signaling pathway, which plays a central role in the regulation of the cellular antioxidant response. Naringenin promotes the nuclear translocation of Nrf2, thereby inducing the transcription of antioxidant enzymes such as HO-1, NQO1, and SOD. This antioxidant defense mechanism significantly reduces intracellular ROS, stabilizes mitochondrial membrane potential, and restores ATP production, all of which are essential for maintaining neuronal energy balance and preventing oxidative damage associated with AD progression. Equally important is naringenin’s role in enhancing AMPK-mediated autophagy, a cellular degradation process that helps remove aggregated and misfolded proteins. Studies have shown that naringenin upregulates autophagy-related proteins such as Beclin-1, ATG5, and LC3-II, leading to increased autophagic flux. This activation facilitates the clearance of toxic Aβ peptides, which accumulate abnormally in the AD brain and contribute to synaptic dysfunction and neuronal death. By restoring autophagy, naringenin helps maintain protein homeostasis and supports long-term neuronal survival, particularly under stress conditions.

Another important signaling axis modulated by naringenin involves the PI3K/Akt and estrogen receptor pathways, which are central to cell survival and anti-apoptotic regulation. Naringenin stimulates phosphorylation of Akt and its downstream target GSK-3β, thereby inhibiting pro-apoptotic mediators like caspase-3. These actions contribute to the protection of neurons from Aβ-induced toxicity. The interaction of naringenin with estrogen receptor pathways also suggests additional relevance for post-menopausal populations, which are at greater risk for AD due to estrogen decline and increased oxidative stress. Furthermore, anti-inflammatory activity constitutes a significant aspect of naringenin’s neuroprotective profile. Chronic neuroinflammation is a hallmark of AD, often characterized by excessive activation of microglia and sustained release of pro-inflammatory cytokines such as TNF-*α*, IL-6, and IL-1β. Naringenin effectively suppresses NF-κB signaling and inhibits MAPK phosphorylation, reducing inflammatory mediator expression and glial reactivity. This modulation of the brain’s immune environment preserves synaptic stability. In addition, naringenin has been shown to inhibit AChE, the enzyme that degrades acetylcholine. By preserving cholinergic signaling, naringenin supports cognitive functions like attention and memory, which are frequently impaired in AD. Together, these interconnected actions—antioxidant defense, autophagy activation, apoptosis inhibition, inflammation suppression, and cholinergic enhancement—highlight naringenin’s potential as a comprehensive therapeutic agent for AD.
